# Robust cell particle detection to dense regions and subjective training samples based on prediction of particle center using convolutional neural network

**DOI:** 10.1371/journal.pone.0203646

**Published:** 2018-10-10

**Authors:** Kenshiro Nishida, Kazuhiro Hotta

**Affiliations:** 1 Department of Electrical and Electronic Engineering, Graduate School of Science and Technology, The University of Meijo, Nagoya-shi, Aichi, Japan; 2 Department of Electrical and Electronic Engineering, Faculty of Science and Technology, The University of Meijo, Nagoya-shi, Aichi, Japan; University of Science and Technology Beijing, CHINA

## Abstract

In recent years, finding the cause of pathogenesis is expected by observing the cell images. In this paper, we propose a cell particle detection method in cell images. However, there are mainly two kinds of problems in particle detection in cell image. The first is the different properties between cell images and standard images used in computer vision researches. Edges of cell particles are ambiguous, and overlaps between cell particles are often occurred in dense regions. It is difficult to detect cell particles by simple detection method using a binary classifier. The second is the ground truth made by cell biologists. The number of training samples for training a classifier is limited, and incorrect samples are included by the subjectivity of observers. From the background, we propose a cell particle detection method to address those problems. In our proposed method, we predict the center of a cell particle from the peripheral regions by convolutional neural network, and the prediction results are voted. By using the obvious peripheral edges, we can robustly detect overlapped cell particles because all edges of overlapping cell particles are not ambiguous. In addition, voting from peripheral views enables reliable detection. Moreover, our method is useful in practical applications because we can prepare many training samples from a cell particle. In experiments, we evaluate our detection methods on two kinds of cell detection datasets. One is challenging dataset for synthetic cells, and our method achieved the state-of-the-art performance. The other is real dataset of lipid droplets, and our method outperformed the conventional detector using CNN with binary outputs for particles and non-particles classification.

## Introduction

In recent years, it is possible to obtain a large amount of cell images. The factors are the development of microscope and cell staining technique. Cell biologists expects to find the cause of pathogenesis by observing the living cells. The number of particles or the density of particles in a cell image is suspected to relation with the cause of pathogenesis.

**Fig 1:**
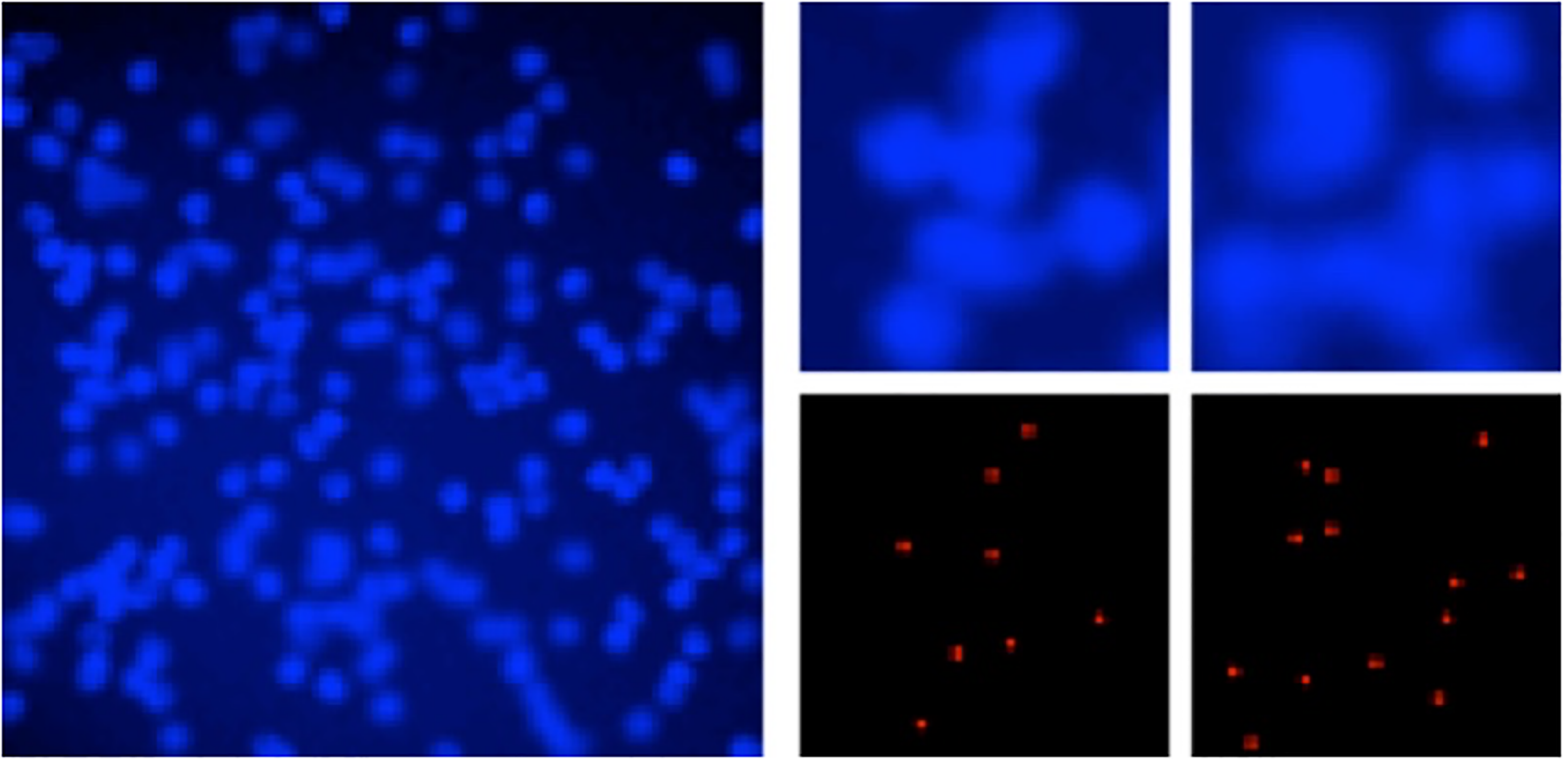
Examples of cell image. Fig 1 shows an examples of cell images. Left image shows the entire cell image. Upper right shows the dense regions of cell image. Lower right shows the ground truth of dense regions.

Examples of cell images are shown in Fig 1. As shown those figures, there are three properties. First, cell images are noisy and low resolution because they captured with high zoom ratio. Second, overlap between cell particles often occurred in dense regions. Third, the characteristic features of cell particles are scanty because many cell particles in cell images are just light spots. These properties make cell image processing difficult. ImageJ [1] is often used in the field of cell image processing. However, ImageJ cannot achieve sufficient accuracy. As a result, human observers need to detect cell particles manually now. Therefore, it takes a lot of time for detecting cell particles, and it cannot handle large amounts of data. In addition, manual detection results are subjective. To elucidate the cause of pathogenesis, a large number of objective detection results are required. Therefore, automatic cell particle detection methods in cell images with high accuracy are required.

Conventional cell particle detection method [2] trains a detector that classifies particles and non-particles, and the detector is applied to a test image by a sliding window search. This approach detects the cell particle with the highest confidence, and the certain region around the cell particle is removed. This process is repeated until the detection candidates are nothing. On the other hand, there are detection methods based on voting such as Hough forest [3] and implicit shape model [4]. Those methods predict the center of an object from multiple parts features, and the prediction scores are voted. Those methods detect the objects with the largest voting value. By predicting the object center from partial views of the object, those methods are robust to partial occlusion.

Convolutional Neural Network(CNN) gave the state-of-the-art performance on various image recognition benchmarks; image classification [5–10], object detection [7, 11, 12], object counting [13], edge detection [14], labeling [15, 16], video classification [17] and so on. CNN mainly consists of convolutional layers, pooling layers and fully connected layers. By the convolutional layer trained by back-propagation algorithm, it allows a deeply powerful feature extraction. In general, CNN requires a large number of training images to set the parameters well. However, we cannot obtain a large number of supervised images because making the ground truth is hard labor for cell biologists.

In this paper, we propose a particle detection method in cell images based on the prediction of cell particle center from the partial views of a cell particle. To predict the center of a cell particle, we use CNN with multiple outputs. Each output corresponds to the distance between the center of a cell particle and the center of an input patch. By voting the prediction results by CNN, the center of a cell particle has high voting values. Our method addresses two kinds of problems in particle detection in cell images. The first is the properties of cell images. Edges of cell particles are ambiguous, and overlaps between cell particles are often occurred in dense regions. It is difficult to detect cell particles by simple detection method using a binary classifier. Our method is robust to overlapping cell particles because our method detects cell particles from peripheral regions. The second is the training samples. The number of training samples for training a classifier is limited, and incorrect samples are included by the subjectivity of the observers. For this problem, our method can easily extract large number of training samples from small number of images because our classifier only predicts the distance to the center of a cell particle by partial views.

In recent years, similar approaches have been proposed [18–20]. However, those papers estimate the offset vector and confidence. In our paper, we estimate only distance from the center of a particle. This is the difference from those papers. If we estimate both distance and direction (offset vector), we can only obtain a single training sample from one particle. On the other hand, if we estimate only distance from the center of a particle, we can obtain many training samples from one particle. Samples on the circumference belong to the same class for training. Since the number of ground truth for cell images is less than that for general objects (e.g. pedestrian, cars, faces), the generation of many training samples from one ground truth is very important.

**Fig 2:**
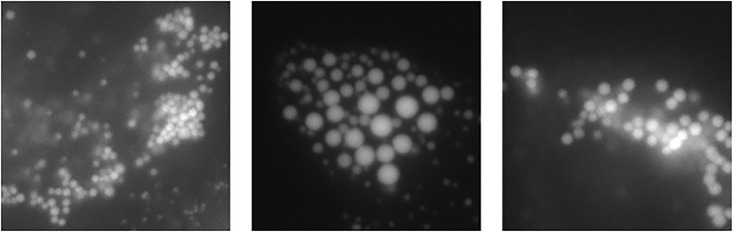
Examples of intracellular image. Fig 2 shows an examples of intracellular images. These cell particles are lipid droplets.

In experiments, we evaluated our method using two kinds of datasets. In the first dataset, the detection targets are synthetic cells [21] as shown in Fig 1. Those images are parts of the challenging dataset for cell detection. In evaluation, our method achieved 96.76% in F-measure. This score outperformed conventional state-of-the-art methods. In addition, we consider that our approach addressed three issues of cell images. In the second dataset, the detection targets are lipid droplets [22] as shown in Fig 2. Evaluation is carried out three times while changing the dataset division. We compare the accuracy by a precision-recall curve and F-measure. Our method achieved 93.9% in F-measure. This score is outperformed the conventional detector using CNN with binary outputs for particles and non-particles classification.

## Methods

**Fig 3:**
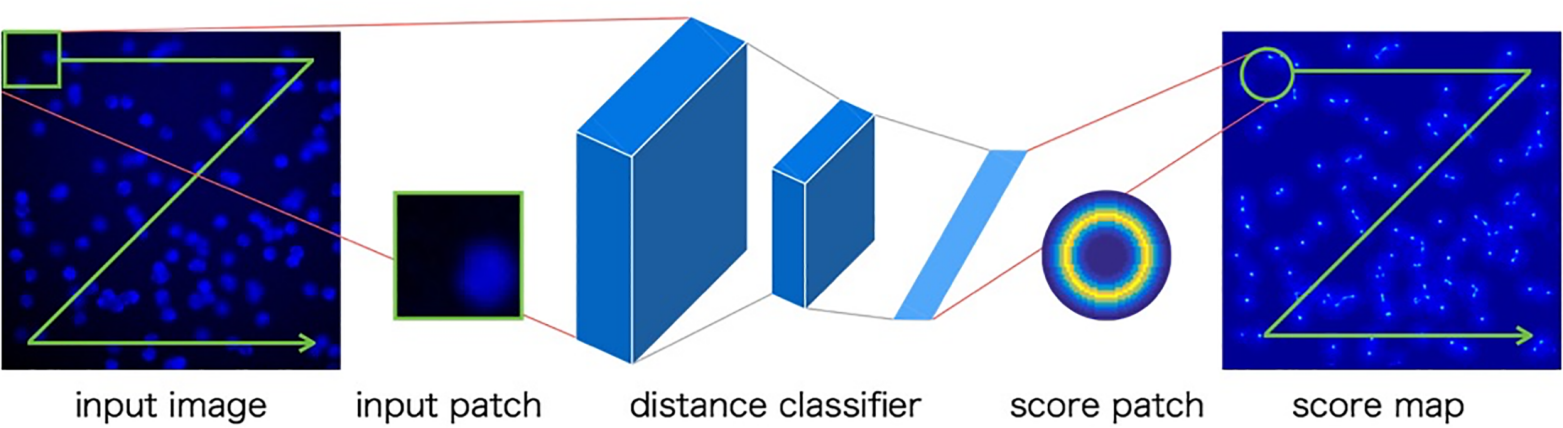
Overview of detection by distance classifier. Fig 3 shows the overview of detection by distance classifier. Our method uses CNN as distance classifier. CNN outputs distance score, and we make a local score patch. We make entire score map by voting those score patches by sliding window search, and detect cell particles.

In cell images, there is overlap between cell particles in dense regions. Some object detection methods are robust to partial occlusion [3, 4] because those methods predict the center of an object from partial views. By voting the predicted center, those methods become robust to partial occlusion. This paper refers to those researches, and we propose a robust cell particle detection method to dense regions using CNN. Overview of the proposed method is shown Fig 3. Our method uses multiple outputs of CNN which predicts the distance between the cell particle center and the center of an input patch. Based on these predicted distances, we make a local score patch. We make entire score map by voting those score patches by sliding window search, and detect cell particles.

### Distance classifier using CNN

**Fig 4:**
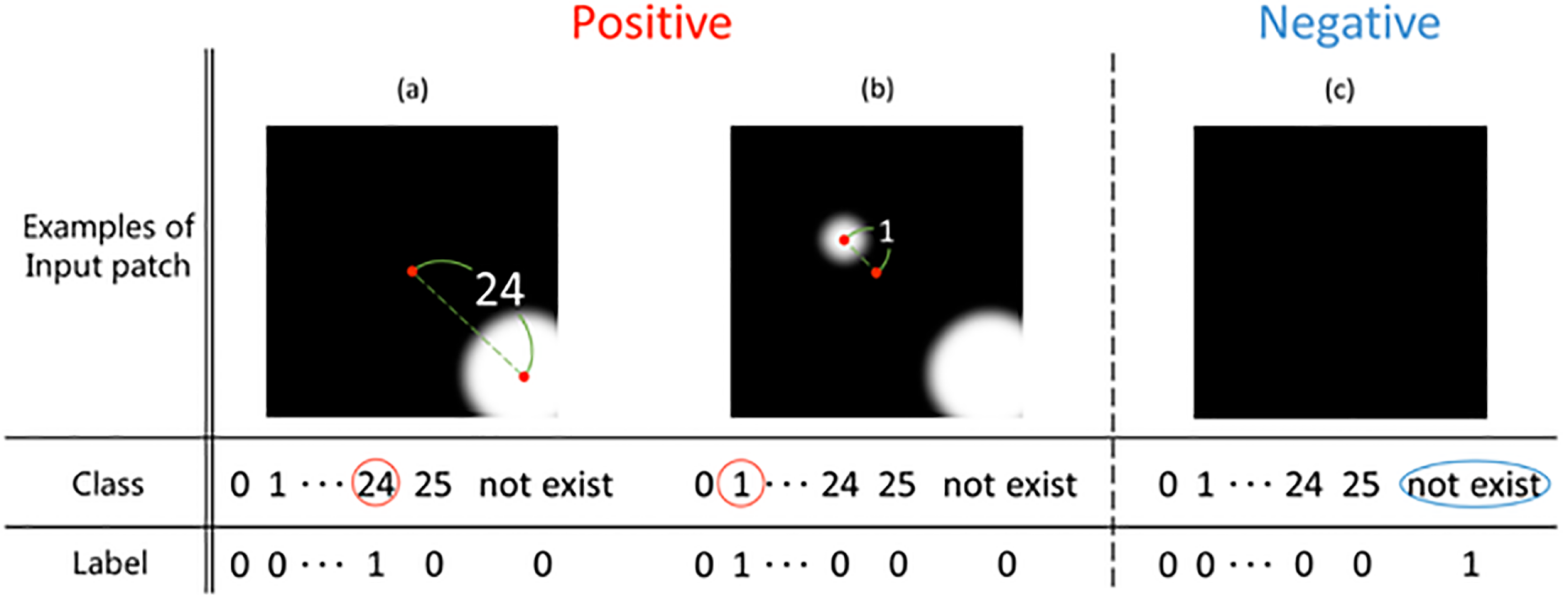
Training patch and class. Fig 4 shows the examples of training patch and class. (a) is the case of input patch including a cell particle. (b) is the case of input patch including multiple cell particles. (c) is the case of input patch that does not including a cell particle.

CNN is trained as a multi-class classifier. We fine tuned the Alexnet architecture [5] for the proposed method. Examples of training patches and classes in our method are shown Fig 4. The top row shows input patches, and a white circle is a cell particle. The lower row expresses training classes for CNN. Those classes mean the distance from the center of the patch to the center of a cell particle. Since the size of a patch is set to 51×51 pixels, the maximum distance from the patch center to the cell particle center is 25 pixels. Therefore, CNN have 26 positive outputs that show the distance from 0 to 25 pixels. Furthermore, we add a negative output, and the number of outputs of CNN is set to 27. If there is a cell particle in the patch, the patch is used as the positive class. For training the positive class, CNN trains the distance from the center of a patch to the center of a cell particle as class. Since the distance is 24 in Fig 4(a), only the class 24 is 1 and others are 0. If there are multiple cell particles in the patch, training cell particle is the nearest cell particle from the center of the patch. Thus, in the case of Fig 4(b), only the class 1 is 1 and others are 0. If there are not any cell particles in the patch, the patch is used as the negative class. For training the negative class that does not include a cell particle center in a patch as Fig 4(c), only the negative class is 1 and others are set to 0. In test phase, the output of our CNN means the confidence score as well as the distance from the center of a patch.

In conventional methods that classify a patch into particle or non-particle class, the number of positive samples corresponds to the number of cell particles in training images. However, in general, the number of supervised cell images is small, and this is a bottleneck in the conventional approaches. Of course, we can use data augmentation that the rotation, shift and scaling of a cell particle are slightly changed. But a cell particle is just a white circle, and the effectiveness of data augmentation is not so large. On the other hand, in the proposed method, we can make multiple training samples from a cell particle because CNN predicts the center of a cell particle from partial view of a cell particle. Thus, our method can make multiple positive samples from near the center of the cell particles. This is also a merit of our method.

### Creating score map by voting score patch

**Fig 5:**
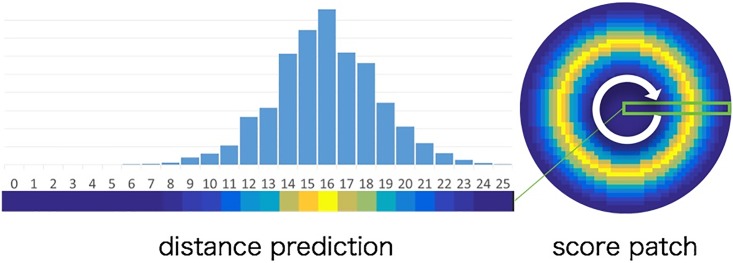
Creating score patch. Fig 5 shows the example of creating a score patch. Left shows distance predictions. Right shows a score patch. Score patch is created for voting. Score patch is circular score distribution made by distance predictions.

We create a score map by scanning the proposed CNN. First, a patch is extracted from a test image, and the patch is fed into the CNN and we get the scores for 27 classes. Next, we create a score patch as shown in Fig 5. We create the score patch by distributing positive scores (0-25 classes) to a circle that corresponds to the distance (0-25). For example, the score for the class 0 is distributed at the center of patch, the score for the class 1 is assigned to a circle with 1 radius. The score patch consists of all scores in the positive class, and the score for the negative class is not used. However, the negative class is functioning because output of our CNN is soft-max layer. Thus, if the cell particles in the patch do not exist, the scores for the positive class became low scores. In contrast, negative class became higher score because the sum of all outputs is 1. The score patch is voted to a test image by sliding window search. By voting all score patches in the test image, we obtain a score map which indicates the confidence of cell particle center.

### Cell particle detection

We count the number of particles in cell images from the score map. To avoid the overlapping detection, we search the region with the maximum score and all scores within the region with circular range are set to negative value. In experiments, we set the radius of a circular region to 25 because the size of a patch is 51×51 pixels. This process repeats until all scores in the map are less than the threshold. We draw precision-recall curve to compute the maximum F-measure. To draw the precision-recall curve, we evaluate the accuracy while changing the threshold. We evaluate true and false detection using the ground truth given by cell biologists. If the ground truth exists within the circular range, we consider that the region is correct detection. On the other hand, if the ground truth does not exist within the circular range, the detection is false positive. If there are multiple ground truths in the circular range, only the cell particle that is near the center is detected and others are treated as false negatives.

**Fig 6:**
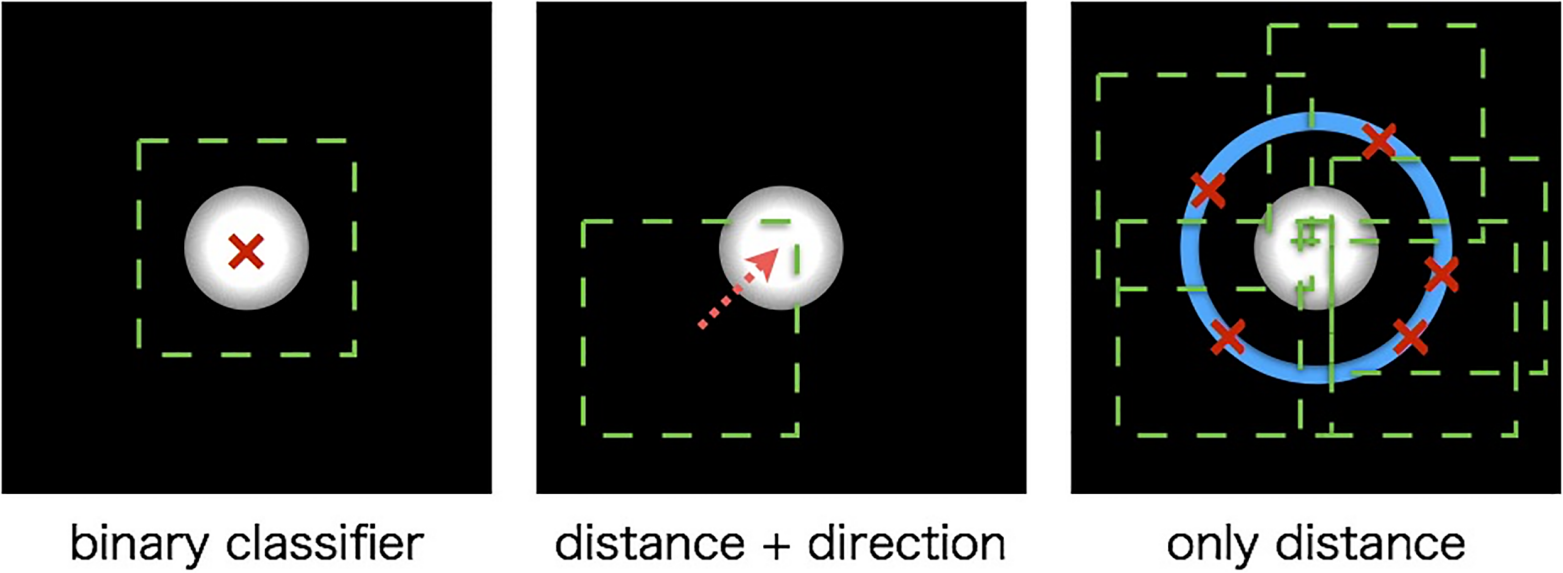
Examples of extracting training samples. Fig 6 shows examples of extracting training samples in three cases of classifier. Left and center are conventional classifier for detection. Right is distance classifier of our proposed method. In extracting training samples of the same class, conventional method can extract one sample from one cell particle. On the other hand, proposed method can extract multiple samples from one cell particle.

By proposed detection method, it is easy to extract may training samples. Fig 6 shows the example of extracting the training samples. In case of a binary classifier, positive samples are cropped from the region where the cell particles are in the center. In case of distance and direction by classifier, training samples are extracted from an area that the distance and direction correspond to the training class. Therefore, training sample of the same class is extracted from only one cell particle in one area. However, the proposed method only predicts the distance from the center of a patch to the center of a cell particle as class. Therefore, the proposed method can extract multiple training samples of the same class from one cell particle.

## Results

In experiments, we evaluate our detection methods on two datasets. First, we experiment with synthetic cell dataset [21]. We compare our proposed method with conventional deep learning method and other small-instance detection approaches [23, 24] which gave the state-of-the-art accuracy. Second, we experiment with lipid droplets dataset [22] which is real data. We compare the proposed method with the conventional detection method using CNN which classifies particles and non-particles.

### Experimental setting for synthetic dataset

In this experiment, we use the synthetic cell dataset [21] which includes 200 cell images. The average number of cells in an image is 171±64. Partial occlusion and image saturation, which are common for fluorescence cell microscopy images, blur at cell boundaries by merging several cells into one group. The resolution of the image is 256×256 pixels. The size of a feature map in CNN becomes small when the number of layers becomes large. Thus, we enlarge the image size from 256×256 pixels to 1000×1000 pixels. In [21], the first 100 cell images are used for training, and the second 100 images are used for testing. However, we use only the first 5 images for training because our method obtains many training samples easily. The size of training patches is 51×51 pixels. Positive patches are cropped from training images. In positive patches, the distance between the patch center and the cell particle center is from 0 to 25 pixels. Thus, the number of positive classes is 26, and each class indicates the distance from 0 to 25. In contrast, negative patches are cropped from training images so that a patch does not contain the center of a cell particle. The negative class has only 1 class, and it indicates the absence of cell particles. Thus, the total number of outputs in our CNN is 27. CNN is applied to patches in a test image and we create a score map based on voting of CNN outputs. By using the created score map, we can detect cell particles.

### Experimental result on synthetic dataset

Table 1 shows the comparison result of our proposed method, detection using CNN as a binary classifier, and conventional state-of-the-art method [21, 24] based on F-measure. Ntrain represents the number of train samples. In case of Ntrain:5, we use 5 images (101-105). We extracted 15,028 positive samples from 5 images. The 15,028 negative samples are extracted that are equal to that of positive samples. In case of Ntrain:1, we use 1 image (101). We extracted 25,168 positive samples. The 25,168 negative samples are extracted. Note that data augmentation is used. Additionally, we use validation set. The number of validation samples is the same as training samples. It is ideal that classifier is applied to all pixels (stride:1) in a test image. However, the computational cost is high because the resolution of image is 1000×1000 pixels. Therefore, our method is applied to pixels and at the interval of 5 pixels (stride:5). The comparison method using CNN as a binary classifier for evaluation. In the comparison method using CNN as a binary classifier, positive samples for training are gathered from the center of a cell particle. Therefore, the number of positive samples is limited up to the number of cell particles. Since the number of training samples is much smaller than the proposed method, we use data augmentation for adjusting the number of training samples. Thus, the number of training samples in the comparison method is the same as our method. On the other hand, we used the patches around the centroid as negative samples to be the same number of training images with our method. We cropped patched randomly, and if the patch includes particle center, the patch is used as a positive sample. If the patch did not contain particle center, the patch is used as a negative sample. In addition, we also compare our method and other small instance detection approaches [21, 24] which achieved the state-of-the-accuracy.

**Table 1:** Experimental results of detection in cell dataset.

	V.Lempitsky [21]	C.Arteta [24]		CNN binary classifier (stride = 1)	CNN distance classifier (stride = 5)		CNN distance classifier (stride = 1)
*Ntrain*	32	32	5	5	1	5	5
*F* − *measure*[%]	94.60	93.67	92.06	95.35	95.26	95.62	96.76

Table 1 shows the experimental results of detection in cell dataset. The two methods on the left are conventional methods, the others are our method. Ntrain represents the number of train samples.

Firstly, we compare our method with the conventional state-of-the-art methods. As shown Table 1, the proposed method achieves the state-of-the-art accuracy. Even if we use only 1 training image, our method gave 0.66% higher accuracy than region-SVM [21] using 32 images for training.

**Fig 7:**
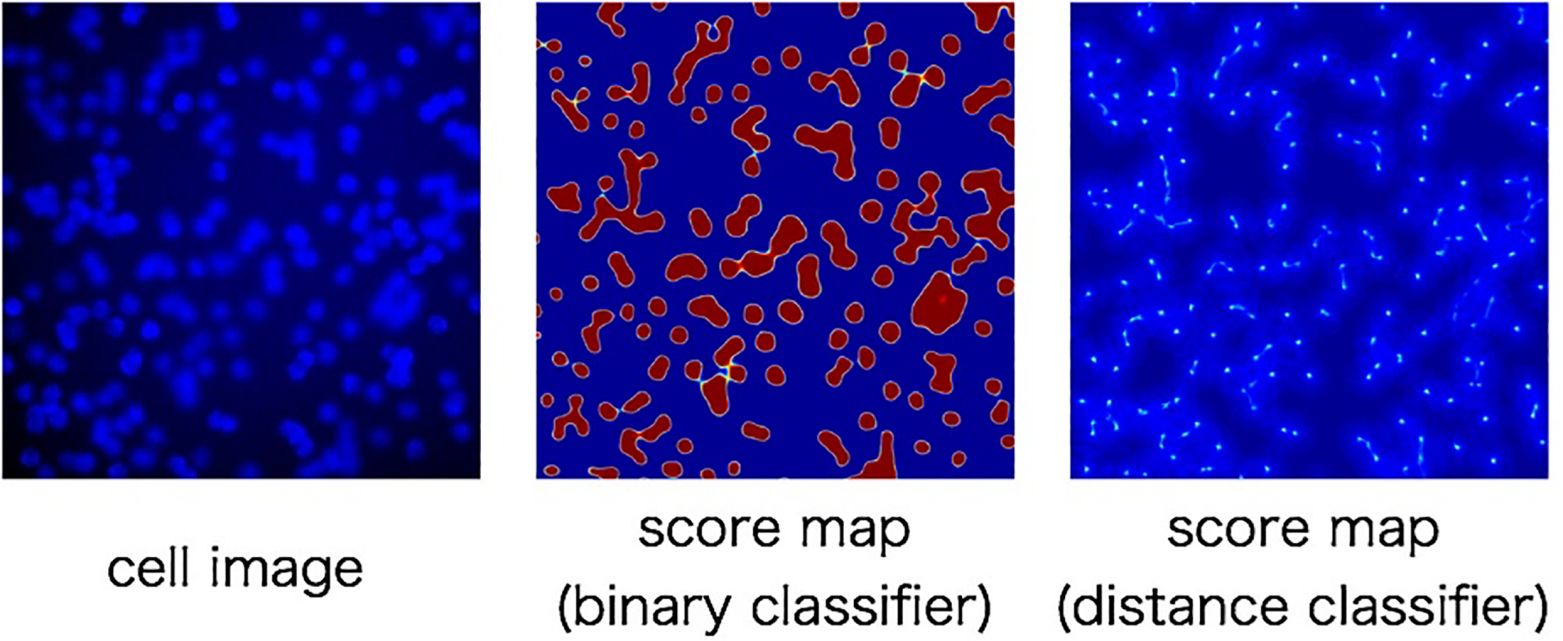
Examples of score map(synthetic cell). Fig 7 shows examples of score map. Left is cell image. Center is score map made by binary classifier. Right is score map made by distance classifier.

Secondly, when we use 5 training images and the stride is 5 pixels, the accuracy of our method has improved to 95.62%. Moreover, when classifier is applied to all pixels, the accuracy has further improved to 96.76%. In comparison with detection using CNN as a binary classifier, our method is 1.41% higher accuracy. The reason why the proposed method achieves higher accuracy is considered to be the robustness to dense regions. To verify the hypothesis, we compare the properties of score map that made by the proposed method and the binary classifier in Fig 7. In case of predicting cell particles using CNN as a binary classifier, there are high scores for the regions including cell particles. Pooling is effective to obtain location invariance. Thus, high score is obtained in the range of several pixels around the center of a cell particle. This makes cell particle detection difficult because there is no boundary between cell particles. On the other hand, our proposed method predicts the center of cell particles using CNN as distance classifier. Fig 7 show that, our method gave a high score at only the center of each cell particle. Moreover, it is robust to overlapping cell particles by voting from peripheral regions. From the above comparison, proposed method can obtain high score at the center of cell particles. Therefore, it enables robust detection to dense regions.

Finally, we show additional experiments to confirm the decrease in accuracy by the error in training data. As mentioned in the previous section, the training data given by the experts may contain errors of several pixels. However, the proposed method can reduce the influence even if training data include errors by voting multiple predictions. To verify the hypothesis, we train the classifier using data with random errors and we compare the decrease in the accuracy.

**Fig 8:**
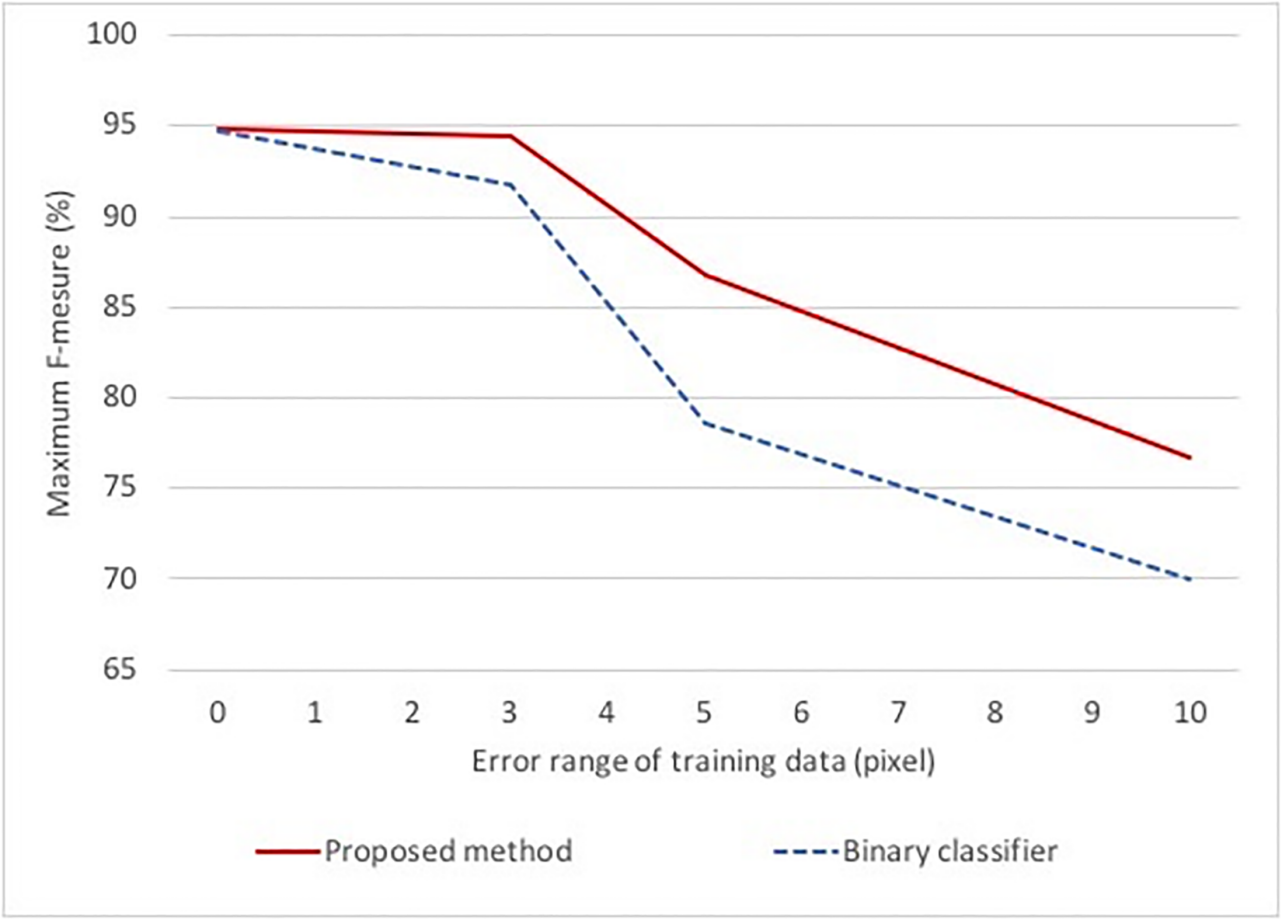
Verification of accuracy degradation by error-containing teacher data. Fig 8 shows the comparison of the decrease in F-measure. Two methods are trained by using the data whose ground truth is randomly changed.

Fig 8 shows the experimental results. The vertical axis shows the maximum F-measure and the horizontal axis shows the range of error given to training samples. When we focus on the F-measure in both detection methods, the F-measure decreases as the error range becomes large. However, the proposed method achieves higher accuracy in all cases than the binary classifier. When the error range is 10 pixels, accuracy decrease of the binary classier was 26.0%. On the other hand, the proposed method was 20.7%. From the above results, even if errors are included in the ground truth, the influence for the proposed method is smaller than the binary classifier.

### Experimental setting for lipid droplet dataset

In this experiment, we performed a similar experiment using real images. The real images are intracellular images of lipid droplets. We use 99 intracellular images with the ground truth position by cell biologists. The resolution of the intracellular image is 200×200 pixels, we enlarge to 1000×1000 pixels.

The size of training patches is 51×51 pixels. Positive patches are cropped from training images that the distance between the patch center and the cell particle center in the patch is from 0 to 25 pixels. Negative patches are cropped from training images so that a patch does not contain the center of a cell particle. Therefore, the number of positive classes is 26, and each class indicates the distance from 0 to 25. On the other hand, the negative class has only 1 class, and it indicates the absence of cell particles. Thus, the number of outputs in our CNN is 27.

We have 99 supervised intracellular images. In experiments, 80 images are used for training, 9 images are used for validation and 10 images are used for test. We evaluated three times while changing the image division. We evaluate the accuracy by using a Precision-Recall curve and maximum F-measure.

In the comparison method, positive samples for training are gathered from the center of a cell particle. Therefore, the number of positive samples is limited up to the number of cell particles. Since the number of training samples is smaller than the proposed method, we use data augmentation for adjusting the number of training samples. The number of training samples is the same as our method.

### Experimental result on lipid droplet dataset

Table 2 shows the comparison result of our proposed method and detection using CNN as a binary classifier. We extracted number about 155,000 positive samples. The number of negative samples is about 155,000 which is equal to that of positive samples. Note that data augmentation is not used. Additionally, we use 55,000 samples as validation set. The parameters of our method are determined by using the accuracy for the validation set. When creating score map, we applied classifier to all pixels in a test image.

**Table 2:** Experimental results of detection in lipid droplet dataset.

	CNN binary classifier	CNN distance classifier
*F* − *measure*[%]	91.43	93.73

Table 2 shows the experimental results of detection in lipid droplet dataset. The F-measure is the average of three times evaluation results.

In Table 2, the F-measure of our method is 2.3% higher than the conventional method. The proposed method uses the voting from partial views to detect a cell particle. Our method is more robust to overlapping cell particles in a dense region, it is also effective for real image.

**Fig 9:**
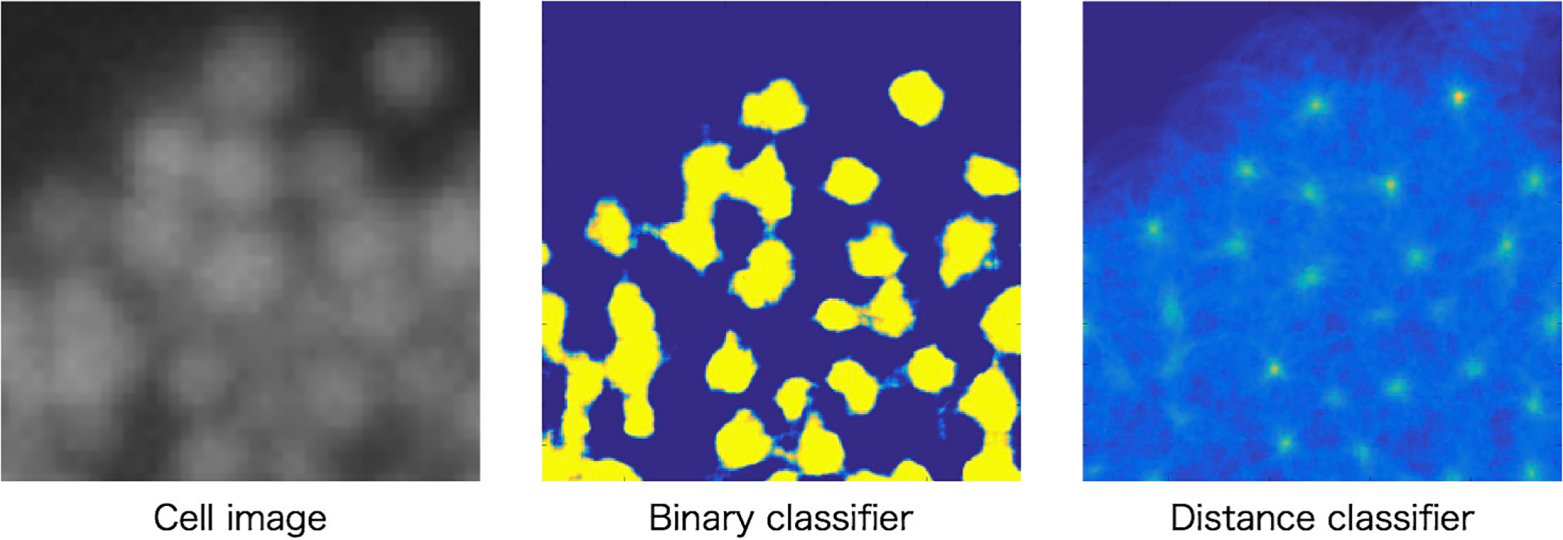
Examples of score map(lipid droplet). Fig 9 shows examples of score map. Left is intracellular image. Center is score map made by binary classifier. Right is score map made by distance classifier.

Fig 9 shows the examples of score maps obtained by our method and the conventional method. In the conventional method, it is mostly classified as binary, and there are high scores around the cell particles because CNN has shift invariance. In contrast to it, our method gave high score to only the center of the cell particles. This is because our method votes the predicted distances obtained by CNN.

## Conclusion

In this paper, we proposed the cell particle detection method based on voting the predicted distance by CNN of partial views. Proposed methods achieved higher accuracy than method using binary CNN and conventional state-of-the-art method. We consider that the reason is addressing the specific problems to cell images.

First, proposed method can extract a larger amount of training samples than conventional method. Conventional method can extract one training sample of the same class from one cell particle. However, the proposed method only predicts the distance. By simplifying this classification task, the proposed method can extract multiple training samples of the same class from one cell particle. Since our method uses deep learning which requires a large amount of learning images, this advantage is useful in terms of practical applications.

Secondly, the proposed method is robust to overlapping cell particles. Conventional CNN classified particles and non-particles, and CNN gave the high score when there is a cell particle at the center of a patch. However, CNN also gave the high score when a cell particle is slightly shifted in a patch. This is because CNN has shift invariance by pooling. Therefore, high score is distributed in the range of several pixels. On the other hand, the proposed method predicted the center of a cell particle. In comparison of two kinds of score maps, the proposed method has a peak for the center of each cell particle.

Thirdly, proposed method is less susceptible to incorrect training data. Since the conventional method calculated a score by binary CNN, only one score is obtained at each location in a test image. Therefore, if one prediction fails, detection is affected. In contrast, the proposed method calculated 26 outputs for a patch and votes the confidence score. In addition, the voting of each location is carried out many times from the various partial views. Therefore, even if one prediction fails, the influence on detection is small. This hypothesis was proved by experiment using artificial error of ground truth locations.

Proposed method is more effective for detection in a dense region. This property is superior to the conventional method but we consider that future work is remaining. It is the improvement of the voting range. Proposed method voted in a circular range with radius 25 pixels because we predict only the distance and do not predict the direction. Therefore, the high value is voted to all directions. This has a merit and demerit. Current method avoids the prediction error of cell particle direction. This is the merit but the proposed method tends to provide higher score in a dense region than a sparse region. This means that cell particles in a sparse region have relatively low score. Thus, we consider that rough direction should be predicted to prevent unnecessary votes. This is a subject for future works.
